# Point-of-care microvolume cytometer measures platelet counts with high accuracy from capillary blood

**DOI:** 10.1371/journal.pone.0256423

**Published:** 2021-08-26

**Authors:** William M. Dickerson, Rebecca Yu, Helena U. Westergren, Jonathan Paraskos, Philipp Schatz, Anna Tigerstrom, Anna Ekman, José Sánchez, Jamie Cheng, Lillian Li, Eugene Y. Chan

**Affiliations:** 1 rHEALTH, Bedford, Massachusetts, United States of America; 2 Precision Medicine & Biosamples, R&D, AstraZeneca, Gothenburg, Sweden; 3 Precision Medicine & Biosamples, R&D, AstraZeneca, Cambridge, United Kingdom; 4 Early Biometrics and Statistical Innovation, Data Science & AI, BioPharmaceuticals R&D, AstraZeneca, Gothenburg, Sweden; 5 DNA Medicine Institute, Bedford, Massachusetts, United States of America; The Ohio State University, UNITED STATES

## Abstract

**Background:**

Point-of-care (PoC) testing of platelet count (PLT) provides real-time data for rapid decision making. The goal of this study is to evaluate the accuracy and precision of platelet counting using a new microvolume (8 μL), absolute counting, 1.5 kg cytometry-based blood analyzer, the rHEALTH ONE (rHEALTH) in comparison with the International Society of Laboratory Hematology (ISLH) platelet method, which uses a cytometer and an impedance analyzer.

**Methods:**

Inclusion eligibility were healthy adults (M/F) ages 18–80 for donation of fingerprick and venous blood samples. Samples were from a random N = 31 volunteers from a single U.S. site. Samples were serially diluted to test thrombocytopenic ranges. Interfering substances and conditions were tested, including RBC fragments, platelet fragments, cholesterol, triglycerides, lipids, anti-platelet antibodies, and temperature.

**Results:**

The concordance between the rHEALTH and ISLH methods had a slope = 1.030 and R^2^ = 0.9684. The rHEALTH method showed a correlation between capillary and venous blood samples (slope = 0.9514 and R^2^ = 0.9684). Certain interferents changed platelet recovery: RBC fragments and anti-platelet antibodies with the ISLH method; platelet fragments and anti-platelet antibodies on the rHEALTH; and RBC fragments, platelets fragments, triglycerides and LDL on the clinical impedance analyzer. The rHEALTH’s precision ranged from 3.1–8.0%, and the ISLH from 1.0–10.5%.

**Conclusions:**

The rHEALTH method provides similar results with the reference method and good correlation between adult capillary and venous blood samples. This demonstrates the ability of the rHEALTH to provide point-of-care assessment of normal and thrombocytopenic platelet counts from fingerprick blood with high precision and limited interferences.

## Introduction

Point-of-care diagnostics deployed in local medical facilities or in-field/home offer advantages of being able to deliver faster results without the need for specimen transport or undue hardship of a patient having to travel to a medical facility. The International Council for Standardization in Haematology (ICSH) stated the increasing need for diagnostics to perform point-of-care full blood counts and published guidelines for their application [1]. While fingerprick blood testing is not new for common analytes like glucose [2], it is less common for cells, like platelets. Fingerprick platelet counts can be performed in a medical PoC setting, however fingerprick blood testing suitable for home use is not available. This has the potential to improve the management of patients receiving vaccines to SARS-CoV-2 [3], drugs which could induce immune thrombocytopenia [4] or platelet disorders [5].

In order for fingerprick blood samples to have utility for point-of-care diagnostics it must first be proven that the sample properties and sampling method is accurate. Studies comparing fingerpick and venous blood counts have shown a variety of results. Schalk et al. 2007 showed fingerprick samples had higher white blood cell count (WBC), higher red blood cell count (RBC), and equal platelet count (PLT) [6]. Hollis et al., 2012 showed equivalent WBC counts [7]. Randels et al. 1997 showed lower fingerprick platelet counts [8]. Furthermore, blood cell counts may show large variability for each drop of blood acquired [9]. These studies taken together imply that sampling method for blood cell analysis from fingerprick greatly affect its utility. Nevertheless, there are successful cases of fingerprick blood being used to do blood cell analysis in medical settings. For fingerprick and venous sample comparisons (N = 189), the BD FACSPresto^™^ demonstrated an R^2^ = 0.973 for absolute CD4 counts and 0.935 for hemoglobin [10]. HemoCue^™^ WBC DIFF had no differences (p = 0.105) in paired samples (N = 8) [11], and the Hemoscreen^™^ has a 510k clearance for fingerstick and venous complete blood counts [12].

Platelet count, especially in the setting of thrombocytopenia, is challenging to measure accurately with central laboratory analyzers and even more challenging at the point-of-care. In a study by Segal et al. 2005, they found that all central lab analyzers except the CELL-DYN (which uses impedance and CD61 fluorescence), overestimated the platelet count in the context of severe thrombocytopenia [13], when compared with the International Society of Laboratory Hematology (ISLH) gold-standard flow cytometry platelet method [14]. The ISLH method relies on two central lab instruments, a cytometer to count CD41/CD61-tagged platelets and an impedance analyzer to provide a reference RBC concentration. At PoC medical settings, the impedance-based CLIA-waived XW-100 does not report platelet values below 100K/μL. The pocH-100i does report down to 10K/μL and uses fingerprick samples, but like the XW-100, it is large (> 15 kg).

In this report, the rHEALTH^®^ ONE technology to perform platelet counts will be introduced. The rHEALTH utilizes a microvolume consumable [15] that holds between 5–10 μL of sample in a capillary format. This sample is loaded in-line with a fluidics tubing that seals on both ends of the capillary to form fluid seals [16]. The sample loader precisely delivers the entire fixed microvolume into the instrument, allowing absolute volumetric sample analysis. After delivery, the sample is analyzed by a compact, palm-sized optical block that performs flow cytometry [17]. The instrument is 1.5 kg, USB-powered, and highly portable [18]. Prototypes of the sample loader and the optical block were tested in microgravity on an aircraft flying parabolic arcs [19].

The purpose of this investigation was to establish the use of the rHEALTH technology, which uses the ISLH CD41/CD61 antibody-labeling approach, for performing platelet counts using fingerprick blood samples. The study had the three specific aims and success criteria. First, concordance in platelet counts to gold standard flow cytometry method. Second, precision < 12.5% at 75k plts/μL in the presence of interferents. Third, the total allowable error (TEa) [20] < 25% at 75k plts/μL.

Complete data sets including both venous and fingerprick blood samples were generated from a total of 31 human subjects. To assess the performance of the rHEALTH vs our ISLH method run on a Coulter brand cytometer (Coulter ISLH), data were analyzed by linear regression, Bland-Altman plots and distribution of percent coefficient of variation (%CV).

## Material and methods

### Recruitment

We enrolled 41 healthy M/F volunteers in year 2018, at the DNA Medicine Institute (Cambridge, MA), from a stated inclusion range between 18–80 years of age, under NASA Internal Review Board Proc0485. The authors confirm that this specific study was reviewed and approved by NASA’s Institutional Review Board Proc0485 before the study began. Volunteers with self-reported infectious disease were excluded. Four people did not have adequate fingerprick blood volume and three did not have adequate venous blood and three were excluded due to rHEALTH instrument error. This resulted in 31 complete sample sets. Written informed consent was obtained in accordance with the International Conference on Harmonization Good Clinical Practice guidelines.

### Sample size and power calculation

The sample size calculation is based on a paired t-test for equivalence between venous and capillary blood platelet count. Using estimates of mean values and variability for capillary and venous blood platelet counts [14], the correlation between the venous and capillary measurements set to 0.8, an equivalence margin of 10%, a significance level of 5%, an assumption of a log-normal distribution for platelet count, N = 31 subjects will achieve a power of 95%.

For the interference studies, the sample size is based on a two-sided test following EP07A2 guidelines assuming normal distribution of the measurement errors:

n=2z1-α2+z1-βs/dmax2

- z1-α2 is the percentile from the standardized normal distribution corresponding to the confidence level 100(1-α) % for two sided-test.
- *z*_1−*β*_ is the percentile from the standardized normal distribution corresponding to the power 100(1-β)%.
- *s* is the repeatability standard deviation of the measurement procedure.
- *d*_*max*_ is the maximum allowable interference to be detected at the analyte test concentration.

We set *d*_*max*_ to 12.5% at 75k plts/μl = 9.3k plts/μl according to CLIA ‘88 grading limits [14], at the 95% confidence level (α = 0.05) and 95% power (β = 0.05). Based on previous experiments a %CV of <10% was assumed and the repeatability was set to 7.3k plts/μl, thus the sample size is *n* = 2[(1.960 + 1.645)7.3/9.3]^2^ = 16.1.

TEa = %CV_APS_*1.65 + B_APS_, where APS = analytical performance specification [20]. At 75k plts/μL and a %CV = 12.5% and a bias (B) = 4.375%, TEa = 25%. Total error (TE) = %CV*1.65 + B.

### Blood collection

Fingerprick and venous blood samples were collected per CLSI GP42-A6 protocols in Cambridge, MA under the IRB protocol (NASA IRB Pro0485), which was approved prior commencing studies described here. From each volunteer, a single K3EDTA (Greiner Bio-One 4ml vacutainer) venous blood sample was taken. Following the venous draw, a fingerprick sample was taken at time point at least 20 minutes but no more than 30 minutes afterwards. The fingers used were both the index and ring fingers. Hands were warmed in a water bath (35-42C) for 5 minutes prior to drying, sterilization, and lancing with BD Microtainer contact-activated lancet high flow 2.0mm x 1.5mm. The first blood drop was wiped and subsequent drops taken into a Sarstedt Minivette POCT 50μL. The blood was ejected into a 0.5mL tube with 50μL 1x PBS K3EDTA. The sample was pipette mixed immediately and again prior to analysis on our Horiba ABX Micros 60 impedance analyzer (ABX) for obtaining red blood cell counts required for Coulter ISLH.

### Sample preparation for rHEALTH and Coulter epics XL (Coulter) cytometer analysis

Each dilution step was done by reverse pipetting to minimize errors. This is performed by depressing the pipette beyond the stop point to draw up additional fluid and then ejecting the sample to the stop point to expel the correct volume. Dilution 1 tube was filled with 190μL (venous) or 180uL (fingerprick) of diluent (PBS 0.1%BSA) whereas dilution 2 and 3 was filled with 150μL of diluent. Before the sample dilutions were prepared, the blood was again mixed completely with a 100uL pipette. Using a positive displacement pipette, 10μL (venous) or 20μL (fingerprick) of blood was pipetted into the dilution 1 containing the 190uL (venous) or 180μL (fingerprick) of diluent. Next 50μL was moved from dilution 1 to dilution 2 tubes then to dilution 3. To three additional 1.5mL tubes, 5μL of both a Coulter CD 41-PE (cat #IM1416U) and BD Biosciences CD 61-PE (cat#561912) was added. Then 100μL of dilution 1–3 blood was added. Samples were mixed on an orbital shaker@1000RPM for 15 minutes at room temperature. Following the incubation, 890μL of diluent was added and pipette mixed. 400 μL of this diluted sample was added to a 5 mL tube with 1600 μL diluent and pipette mixed. This represents a 1:1000 dilution of the initial blood sample.

### Analysis of sample on rHEALTH and Coulter

Sample dilutions were split and analyzed on the Coulter flow cytometer and rHEALTH. Results for both instruments were available to the operators. Following the runs, the RBC, RBC doublets, PLT, and both RBC/PLT counts were recorded. A FSC (x-axis) vs. FL-2 (y-axis) was visualized in a scatterplot for quadrant analysis. PLT were found in the upper left quadrant, RBC in the lower right and double-dyed cells with size of a red cell in the upper right-hand quadrant. Signals of RBC doublets were observed in the same quadrant and the number of RBC doublets was recorded separately.

**Fig 1:**
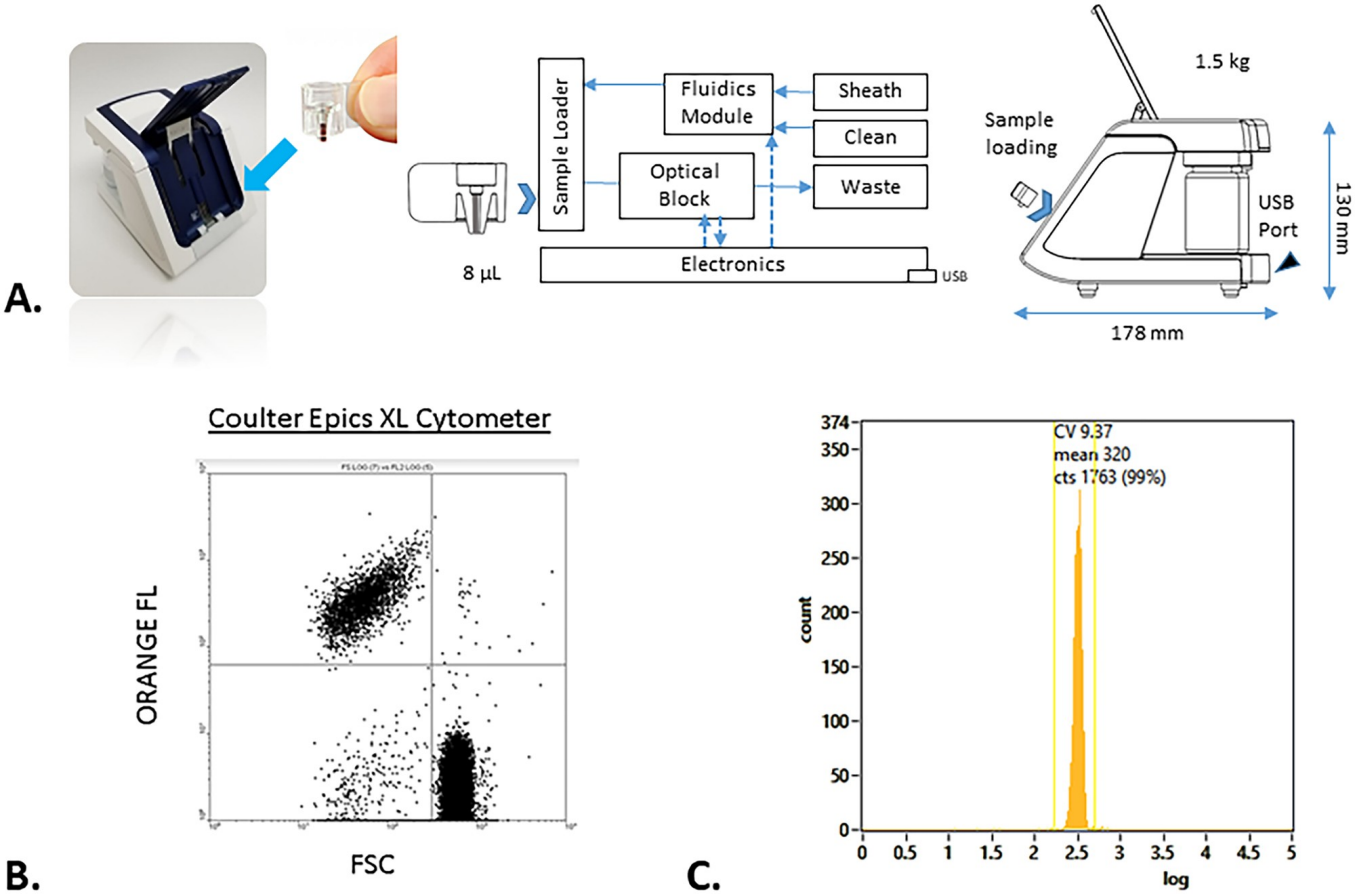
Overview of the rHEALTH method for microvolume cytometry analysis of platelets and data from benchmark cytometer and rHEALTH. **A.***Left*, 8 μl capillary blood sample in a clear plastic consumable is loaded into the instrument for analysis. *Middle*, The sample consumable is received by a plunger-based, in-line sample loader which seals around its two open ends. Pressure (~ 70 mbar) is applied to the system which drives the entire sample volume, via hydrodynamic focusing, into a miniaturized optical module for laser-based cytometry detection. A fluidics module with electronic valves manages fluids from the pressurized sheath and clean bottles (60 cc). The analyzed sample passes through the optical block and into the removable waste bottle (60 cc). The electronics with embedded software manage instrument control and data capture. Dashed lines indicate connection to the optical and fluidics modules. Arrows from the electronics module indicate a control function and arrows to the electronics indicate a data function. The USB provides power, control commands, and data output to and from a PC. *Right*, The device is shown in its side view with dimensions, mass, USB 2.0 port, and sample loading orientation. A PC computer (not shown) provides power (up to 2.5W) via the USB connection, collects raw data, and performs data analysis. **B.** XY scattergraphs generated by Coulter cytometer in quadrant analysis. **C.** rHEALTH histogram analysis of total platelet count includes all orange fluorescent events.

rHEALTH data collection was accomplished by loading 8 μL of each diluted sample using a positive displacement pipette into the consumable, inserting the consumable into the device, and initiating the run (Fig 1A). The in-line sample microvolume sample loader analyzes the entire loaded volume to yield absolute volumetric counts. The fluorescence burst data were collected and analyzed with the rHEALTH VIEWER software. All orange fluorescent events were plotted in a histogram to record the platelet count. Incomplete runs, as indicated by lack of an end bubble during the sample runs, were excluded.

### rHEALTH and Coulter ISLH data calculations

The flow cytometry data were reported according to the orange fluorescence versus FSC quadrant (Fig 1B). The upper left quadrant contained CD41/CD61 platelets. The upper right quadrant contained platelets and RBC. Noise was observed in the lower left and RBC and RBC doublets were observed in the lower right quadrant. To attain the correct RBC count:

$${\text{R}}_{\text{T}}=\text{R}+{\text{C}}_{\text{PR}}+{\text{C}}_{\text{RR}}$$ (1)

where R_T_ = total RBC counts, R = RBC singlet counts, C_PR_ = platelet RBC coincidence events, and C_RR_ = RBC coincidence events. For platelet counts;

$${\text{P}}_{\text{T}}=\text{P}+{\text{C}}_{\text{PR}}$$ (2)

where P_T_ = total platelet count, P = platelet singlet count.

Calculation of total platelet for flow cytometry

$$\left[\text{P}\right]=\left({\text{P}}_{\text{T}}/{\text{R}}_{\text{T}}\right)\left[\text{RBC}\right]$$ (3)

Where [P] = absolute platelets per μL, P_T_ = total platelets, R_T_ = total RBC counts and [RBC] = red blood cell count per μL on clinical analyzer.

For the rHEALTH data were analyzed using histogram analysis for platelets (Fig 1C),

$${\text{P}}_{\text{T}}=\text{P}$$ (4)

Where P_T_ = total platelets and P = total orange events

added up to the total platelet count per microliter of blood sample using absolute volume analysis on the rHEALTH

$$\left[\text{P}\right]={\text{P}}_{\text{T}}\;\text{x}\;\text{DF}/\text{V}$$ (5)

where [P] = absolute platelets per μL, DF = overall dilution factor, and V = volume loaded into rHEALTH.

### Data correlation

Scatterplots between the rHEALTH and Coulter ISLH were produced and linear regression analysis performed (Prism, GraphPad, CA). A Bland-Altman plot was generated based on plotting the difference (or differences as percentages) between the methods versus the average of the methods [21].

### Interferent testing

Potential interferents were tested on prepared thrombocytopenia and hyperlipidemic normal platelet count whole blood samples. Medium and low-level thrombocytopenia samples were prepared with platelet rich plasma replaced with varying amounts of PBS [22]. The interferents spike-in included fragmented red blood cells (0.8million/μL) and platelet lysates (0.2 million/μL), triglycerides 37mg/mL (MilliporeSigma #17811-1AMP), low density lipoproteins (LDL) 3mg/mL (MilliporeSigma #L8292-1VL), Integrin antibodies to alpha2b (R&D Systems #MAB7616) and beta 3 (R&D Systems #AF2266) 15μg/mL each. Additionally, samples were run with the instrumentation cold (4–8°C), room temperature (24–26°C) or hot (25–30°C).

## Results

### Fingerprick method development

A minimum of 50μL of blood was the minimum blood sample necessary to run the platelet assessment via the Coulter ISLH and rHEALTH methods. Multiple lancets were tested including the Surgilance 21 gauge/SLN300, the 18 gauge/SLB 200 and the BD Microtainer contact-activated lancet high flow 2.0mm x 1.5mm. The BD Microtainer contact-activated lancet was selected for sampling throughout the study due to routinely (> 95%) delivering at least 50μL blood volume. Sarstedt Minivette POCT (50 and 100μL) devices were found to collect sample easily and allowed for full volume recovery, while the larger volume BD Microtainer (minimum 250 μL) and the Sarstedt Microvette (100 μL) were hard to fill adequately.

During sample processing it was observed that occasionally cells sedimented to the bottom of the microcentrifuge tube and that residual anti-coagulant remained on the walls of the Sarstedt pipette after sample ejection. Gentle up and down pipetting allowed for the re-suspension of the cells but also introduced bubbles and blood loss to pipette tips. 50μL of blood was dispensed into a tube already containing 50μL of PBS EDTA for mixing without loss of blood and formation of air bubbles, and to ensure proper mixing with anticoagulant.

### Platelet assay standardization

Previously, an assay to perform platelet counts using the rHEALTH was developed, similar to the ISLH method. The ISLH method requires the labeling of platelets with two fluorescently labeled antibodies to the platelet surface marker CD41 and CD61. Subsequently, the samples were diluted to a minimum of 1:1,000 and analyzed on the flow cytometer. The sample were also analyzed on a clinical analyzer to determine the red blood cell counts per microliter of whole blood. The flow cytometry red blood cells number was divided by the clinical analyzer red blood cell concentration to calculate the total whole blood volume used for the flow cytometer. For both methods, the platelet #/microliter concentration was generated by multiplying the dilution factor. The same dilution was used for both analysis methods to reduce variability. To best capture the range of possible platelet counts seen in normal and low platelet populations, three dilutions of blood were included representing (neat, 1:4 and 1:16) which equated to final dilutions of 1:1000, 1:4000 and 1: 16,000.

### Sample blood testing

Samples from 31 volunteers were included in the data analysis. Each sample was taken early in the morning and analyzed within 6 hours, which is within the 24 hours as recommended by ICSH for blood count measurements [23]. Controls and calibrants were analyzed daily on each instrument. The blood samples were brought into the lab and read on the ABX immediately after the sample was acquired. The assay was completed and samples stored in a cooler with a cold pack until analysis on rHEALTH and Coulter.

**Fig 2:**
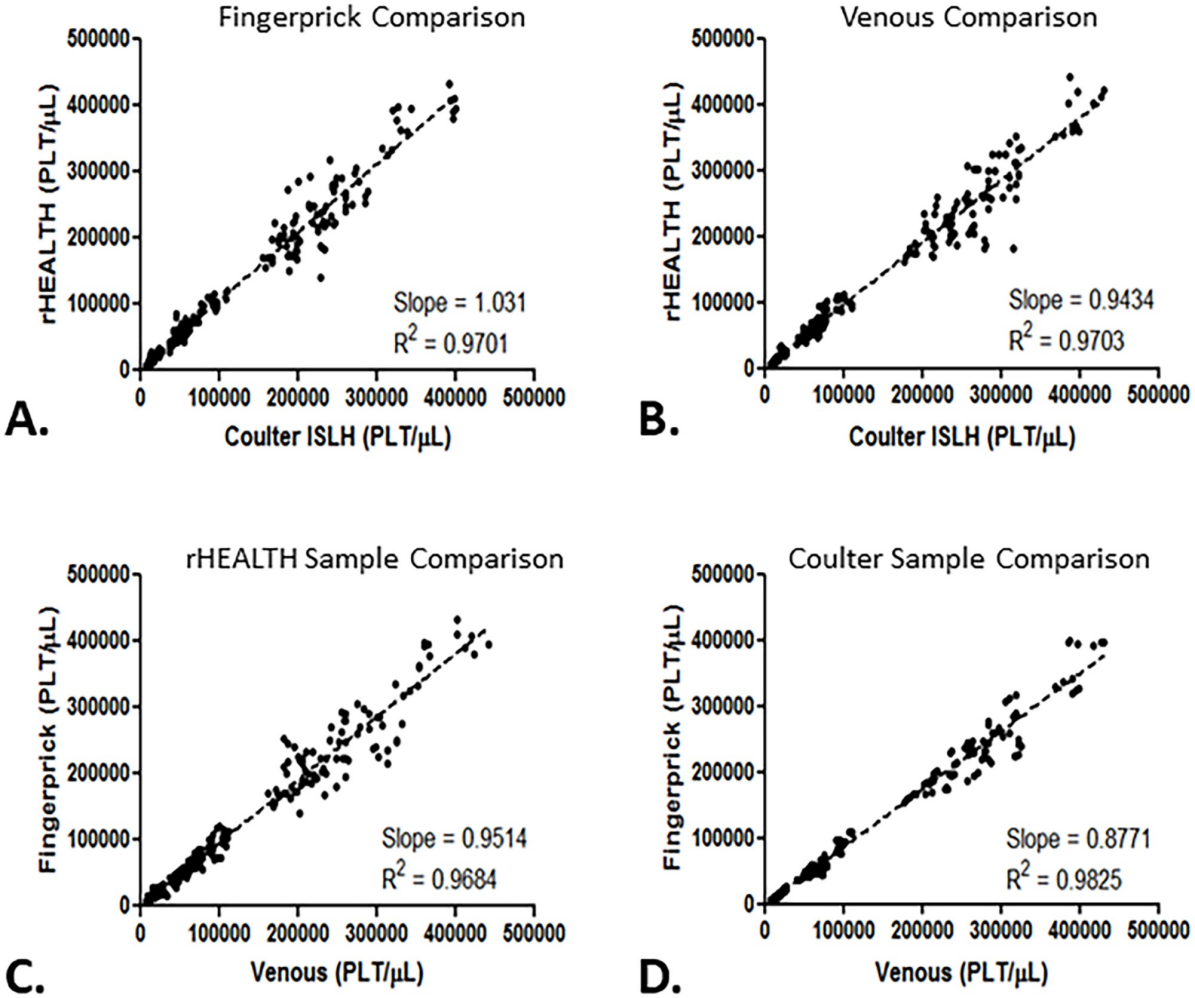
Performance of rHEALTH and Coulter ISLH for fingerstick and venous samples. Plot of rHEALTH absolute volumetric PLT counts versus Coulter ISLH for **(A)** fingerprick samples and **(B)** venous samples. Plot of venous platelet counts versus fingerprick platelet counts using Coulter ISLH and the rHEALTH absolute volumetric PLT for **(C)** rHEALTH and **(D)** Coulter. Platelet concentrations for all are in plt/μL and the trend line equation and R^2^ are shown.

Each data point for the rHEALTH and Coulter was calculated according to the equation above. The values for each sample at each dilution for each of the triplicate measurements is plotted in concordance plots of rHEALTH volumetric count method, versus Coulter ISLH. A total of 279 individual determinations were included in the final data set on the Coulter and 278 on the rHEALTH. One measurement was excluded due to an in-completed run. A linear regression analysis showed a slope of 1.031 and an R^2^ = 0.9701for fingerprick samples (Fig 2A); and a slope of 0.9434 and an R^2^ = 0.9703 for the venous blood samples (Fig 2B). This indicated good agreement between the two methods using orange triggered volumetric counts on the rHEALTH versus Coulter ISLH.

Platelet values for venous blood samples and fingerprick blood from the same individuals are compared with the rHEALTH ONE and Coulter ISLH. To assess the agreement between them, a linear regression analysis was performed. For rHEALTH a slope of 0.9514 and R^2^ = 0.9684 for rHEALTH (Fig 2C) was obtained; similarly, a slope of 0.8771 with a R^2^ = 0.9825 was obtained for Coulter ISLH. Platelet count analyzing fingerprick blood and venous blood correlated well when using the rHEALTH volumetric method.

**Fig 3:**
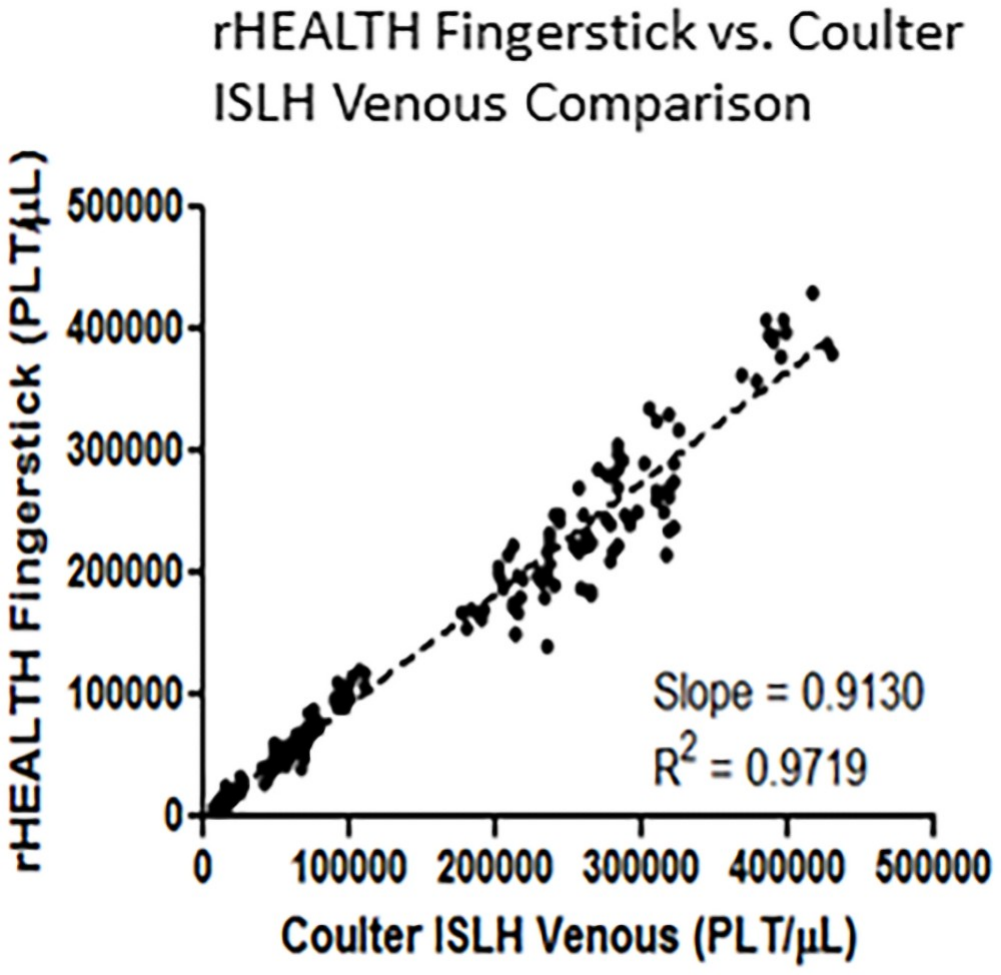
Correlation plot of Coulter ISLH venous platelet counts versus rHEALTH fingerprick platelet counts using absolute volumetric method. Platelet concentrations for both are in plt/μL and the trend line equation and R^2^ are shown.

The platelet count from venous blood draw using Coulter ISLH was compared to the platelet count of fingerprick samples using rHEALTH (Fig 3). A concordance with a slope of 0.9130 and R^2^ = 0.9719 was observed.

**Fig 4:**
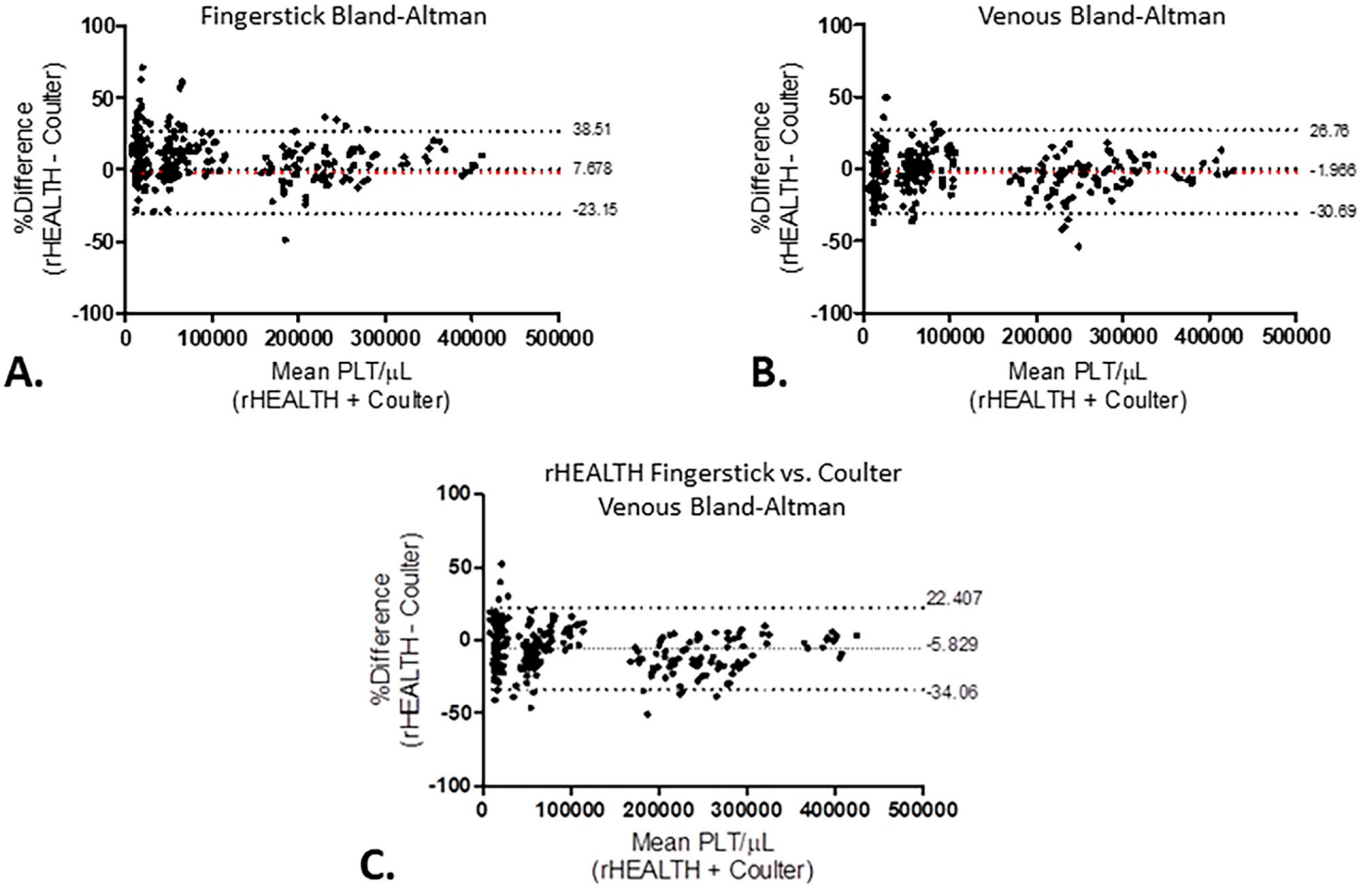
Bland-Altman analysis of the rHEALTH absolute volumetric approach versus Coulter ISLH. The bias is shown with +/- 1.96 SD bounds. **(A)** Fingerprick samples for both approaches, **(B)** Venous samples for both approaches, and **(C)** Bland-Altman of Coulter ISLH venous versus rHEALTH fingerstick analysis.

The bias between the rHEALTH volumetric and Coulter ISLH was assessed by a Bland-Altman analysis. No offset bias of venous samples was detected and approximately 8% higher values for rHEALTH analysis of fingerprick blood samples (Fig 4) compared to Coulter ISLH were found. rHEALTH fingerprick versus Coulter ISLH venous comparison showed a bias of 5.8%, which was between that of the venous and fingerstick biases. Further Bland-Altman analysis comparing all three methods, including ABX, show the smallest bias and 95% intervals between rHEALTH and Coulter ISLH (S1 and S2 Figs).

**Fig 5:**
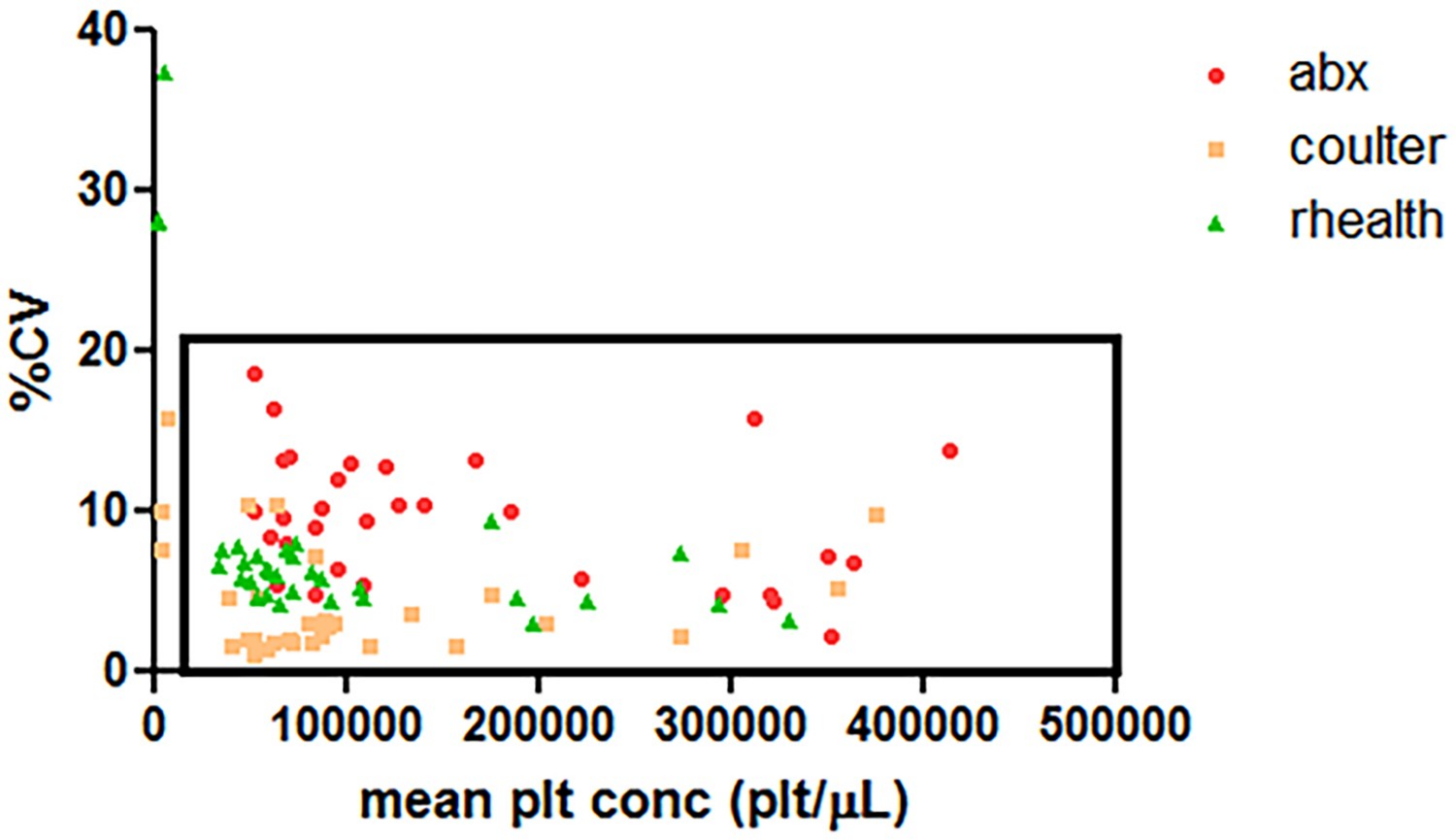
Graphical analysis of the ABX, Coulter ISLH, and rHEALTH precision data. The effect on platelet counts precision (N = 16 per condition). The precision for ABX Micros 60 at 2.2–18.7%, Coulter ISLH at 1.0–15.9 and the rHEALTH at 3.1–37.5% for all samples. Excluding the anti-platelet antibodies, which yielded close to zero counts via Coulter ISLH and the rHEALTH, the precision for non-zero platelet counts, indicated in the boxed area,was as follows: ABX Micros 60 at 4.5–18.7%, Coulter ISLH at 1.0–10.5 and the rHEALTH at 3.1–8.0%.

The effects of interferents listed in Table 1, showing the rHEALTH as the only instrument unaffected (p > 0.05) by RBC fragments. In contrast to the ABX, both the rHEALTH and the Coulter ISLH was unaffected by triglycerides in at least one sample set. As predicted, the ABX was the only instrument unaffected by the negative control anti-platelet antibodies. The mid-thrombocytopenia LDL sample was impacted more than the low-thrombocytopenia sample for both the Coulter ISLH (89.8% vs. 99.7%) and the rHEALTH (81.0% vs. 103.0%) in contrast to the ABX where it was higher for the low sample (153.5% versus 182.9%). The rHEALTH performed the same at room temperature and in the cold (4–8°C), but differently at elevated temperature (25–30°C). When compared with Coulter ISLH, the rHEALTH performed better at elevated temperature for the hyperlipidemia samples (121.2% versus 173.8%), but worse with platelet lysates (127.6–142.2% versus 105.0–109.8%). The %CV for the interference study was plotted versus the mean platelet concentration for the three methods (Fig 5). Excluding the effect of anti-platelet antibodies, the precision for the ABX ranged from 4.5–18.7%, Coulter ISLH at 1.0–10.5%, and the rHEALTH at 3.1–8.0%.

**Table 1:** % of expected PLT concentration with interferents.

Interferent	Concentration	Instrument	Report Mid thrombocytopenia % of expected	Report Low thrombocytopenia % of expected	Report Normal platelet range with hyperlipidemia % of expected
RBC fragments	0.8x10^6^/μL	Coulter	140.4 (<0.001)	132.9 (<0.001)	134.2 (<0.001)
		ABX	221.2 (<0.001)	267.2 (<0.001)	186.3 (<0.001)
		rHEALTH	101.4 (0.447)	102.2 (0.23)	104.5 (0.16)
Platelet lysate	0.2x10^6^/μL	Coulter	105.0 (0.009)	109.8 (<0.001)	105.6 (0.007)
		ABX	115.6 (<0.001)	128.6 (<0.001)	109.7 (0.001)
		rHEALTH	127.6 (<0.001)	142.2 (<0.001)	112.7 (<0.001)
Triglycerides	3700 mg/dL (ref <150 mg/dL)	Coulter	90.1 (<0.001)	87.2 (<0.001)	ND
		ABX	193.0 (<0.001)	191.0 (<0.001)	ND
		rHEALTH	105.0 (0.031)	91.7 (<0.001)	ND
LDL	300 mg/dL (ref <100 mg/dL)	Coulter	89.8 (<0.001)	99.7 (0.868)	ND
		ABX	153.5 (<0.001)	182.9 (<0.001)	ND
		rHEALTH	81.0 (<0.001)	103.0 (0.177)	ND
Anti-platelet Antibodies	30ug/mL	Coulter	3.4 (<0.001)	5.4 (<0.001)	5.3 (<0.001)
		ABX	97.5 (0.423)	86.1 (<0.001)	91.9 (0.004)
		rHEALTH	1.1 (<0.001)	4.4 (<0.001)	3.3 (<0.001)
Low temp	4–8°C	Coulter	ND	ND	ND
		ABX	ND	ND	ND
		rHEALTH	104.7 (0.0932)	97.0 (0.2381)	95.4 (0.0760)
High temp	25–30°C	Coulter	97.6 (0.0933)	97.8 (0.1239)	173.8 (<0.001)
		ABX	101.6 (0.7462)	107.2 (0.0483)	103.6 (0.3602)
		rHEALTH	90.6 (<0.001)	87.0 (<0.001)	121.2 (<0.001)
Summary	Total of each color	Coulter	8	1	7
		ABX	6	2	8
		rHEALTH	11	3	5
**Legend**					
% from expected			Green (± 12.5%)	Yellow (± 25%)	Red (> 25%)

Values listed as percentage of unspiked sample with p-value in parentheses. For instance, 90.6 (< 0.001) would be 90.6% of unspiked sample having a p-value < 0.001 (statistically different than the unspiked).

The concentration or condition of each interferent is listed in the second column, along with the clinical reference ranges (where possible) for adults age ≥ 20. For triglycerides and LDL, very high spike-in values were selected to represent worst case scenarios. The % of expected platelet values with (p-value) are tabulated for each sample, with 100% as ideal. Higher p-values are desirable, indicating no effect of the spiked interfering agent on the sample when compared with its control. Mid-thrombocytopenia samples in whole blood were 70-90k/μL. Low-thrombocytopenia samples in whole blood were 20-30k/μL. The table is color coded with green (G) boxes having values within 12.5% of original, yellow (Y) boxes within 25.0%, and red (R) boxes > 25.0%. For each method, a tabulation of the number of conditions for each color is included.

Based on these interference experiments, TE was calculated based on results from the precision interference study and bias between venous and fingerprick samples. The maximal TE = 20.9%, based on a maximum rHEALTH %CV = 8.0% and maximum bias = 7.7%.

## Discussion

The purpose of this investigation was to establish the use of the rHEALTH ONE technology for performing absolute volumetric platelet counts using point-of-care fingerprick blood samples. The rHEALTH method utilizes 8 μL of sample to perform absolute counting. This study has shown that the platelet count using a fingerprick blood sample is comparable to the venous platelet count and the rHEALTH method can perform platelet counts comparable to the gold standard ISLH method [14], developed to address shortcomings of the impedance only method in measuring thrombocytopenic samples. Since the rHEALTH ONE is a small device, it has the potential to perform measurements at the point-of-care where fingerprick blood samples are readily obtained. In contrast, the ISLH method requires two large instruments in a laboratory setting, which is most compatible with venous blood samples. The ISLH method is the preferred standard method for calibrating platelet counts for reference samples, especially those with thrombocytopenia.

Platelet counts were collected on each individual sample at each of the three dilutions spanning counts from <10,000 to >600,000 plts/μL. The rHEALTH platelet counts were comparable to those using Coulter ISLH method using venous and fingerprick blood samples. Good correlation (R^2^ > 0.90) was observed in all cases of the rHEALTH with Coulter ISLH. This should allow fingerprick blood samples to be used for assessing a patient’s platelet counts. The optimized fingerprick sample collection was important. The choice of lancet was important in getting good sample flow and the mixing of the sample with a saline K3EDTA solution ensured proper anti-coagulation. In the context of interfering substances, the rHEALTH was most reliable, had the best precision, and met our < 12.5% precision success criteria, where only platelet fragments and anti-platelet antibodies substantially interfered with proper recovery of platelet counts. The TE was less than TEa of 25% under the most stringent conditions with high levels of interferents and thrombocytopenic counts, allowing accurate results under simulated disease conditions.

This study shows the ability to have a high-performing, robust PoC platelet test from capillary blood performed using the rHEALTH. Its absolute volumetric counting decreases the need for calibration. The device as tested was a prototype and likely to have optimal performance and features as an engineered product. Addition of a forward scatter size discriminator may help in distinguishing intact platelets from fragmented platelets. For home use, automation of manual dilution and metering steps would be important and may be done as part of the instrument or a consumable with our microgravity-compatible automatic passive microfluidic mixer [19,24]. A battery, embedded data analysis, connectivity, and automated error flagging will further enhance usability and data sharing for patients using the device at home. Addition of a pressurized fluid bladder system, as we have tested on parabolic flights [19], will make the instrument fully operational in zero gravity. The device’s low mass, volume, and power are all 10-fold less than other point-of-care blood analyzers, making it uniquely suitable for space and home, where astronaut crew members and patients in diverse settings can leverage the technology’s good correlation between capillary and venous blood samples for sound clinical decision-making related to normal and thrombocytopenic blood samples.

## Supporting information

S1 Fig Bland-Altman analysis of methods with ABX impedance analyzer for high platelet sample. The bias is shown with +/- 1.96 SD bounds. *Top row*, *left-to-right*. Venous first samples (high point) Bland-Altman plots comparing the three methods. *Bottom row*, *left-to-right*. Fingerstick first samples (high point) Bland-Altman plots comparing the three methods. The medium and low dilution points were not analyzed on the ABX because they were outside the stated protocol for the instrument. Thrombocytopenia is < 1.5x10^5^ PLT/μL and normal range is 1.5x10^5^ to 4.5x10^5^ PLT/μL.
(TIF)

S2 Fig Bland-Altman plots of the interference study between the three methods. The bias is shown with +/- 1.96 SD bounds. *Left-to-right*, Coulter ISLH versus rHEALTH, ABX versus Coulter ISLH, and ABX versus rHEALTH. Thrombocytopenia is < 1.5x10^5^ PLT/μL and normal range is 1.5x10^5^ to 4.5x10^5^ PLT/μL.
(TIF)
