# Supplementary figures and images for: Point-of-care microvolume cytometer measures platelet counts with high accuracy from capillary blood
Source: PLoS One. 2021 Aug 26;16(8):e0256423. doi: 10.1371/journal.pone.0256423 (PMC8389400; doi:10.1371/journal.pone.0256423)

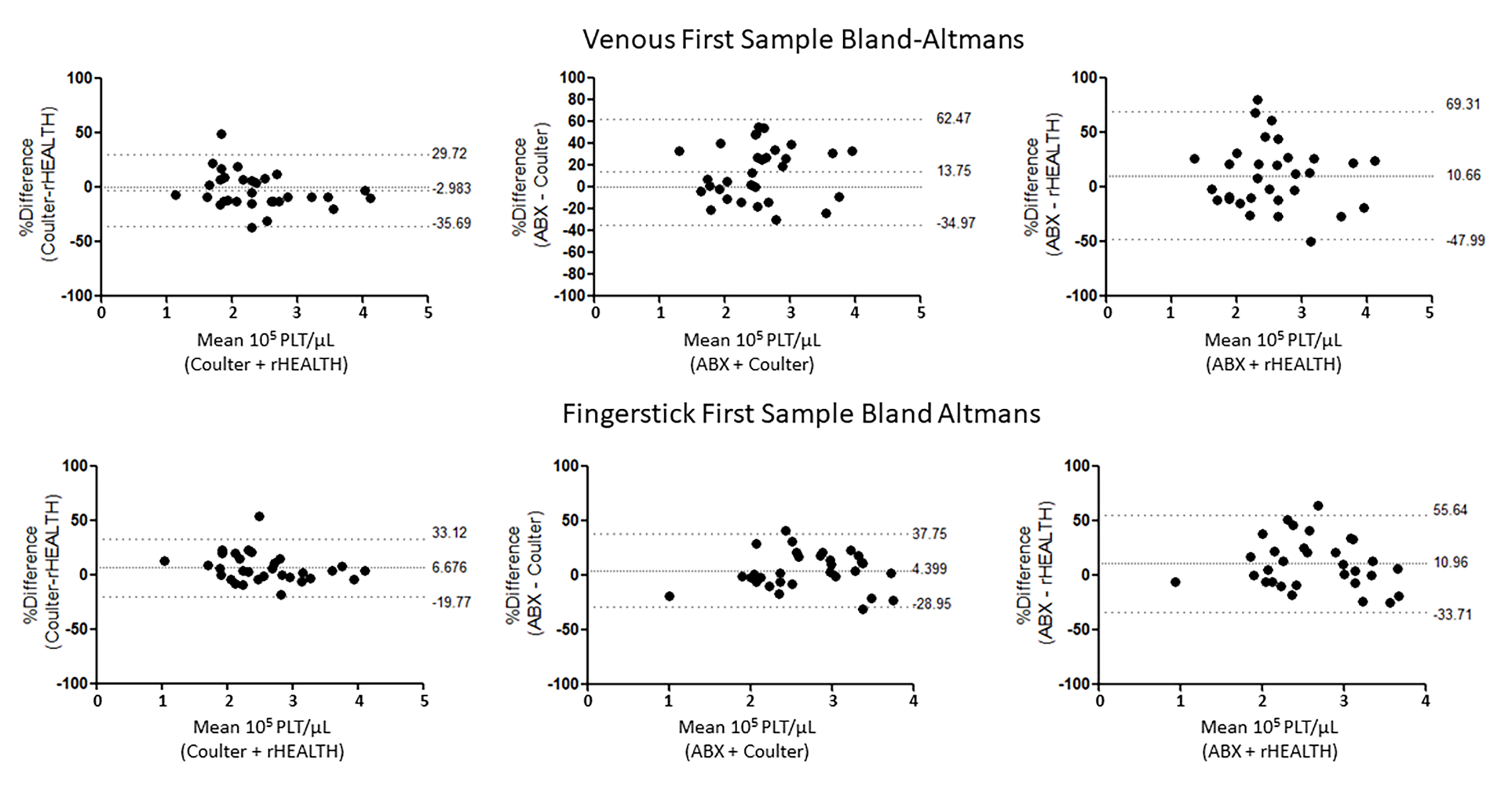

Supplement: S1 Fig — The bias is shown with +/- 1.96 SD bounds. Top row, left-to-right. Venous first samples (high point) Bland-Altman plots comparing the three methods. Bottom row, left-to-right. Fingerstick first samples (high point) Bland-Altman plots comparing the three methods. The medium and low dilution points were not analyzed on the ABX because they were outside the stated protocol for the instrument. Thrombocytopenia is < 1.5x10^5^ PLT/μL and normal range is 1.5x10^5^ to 4.5x10^5^ PLT/μL. (TIF) [file pone.0256423.s001.TIF]

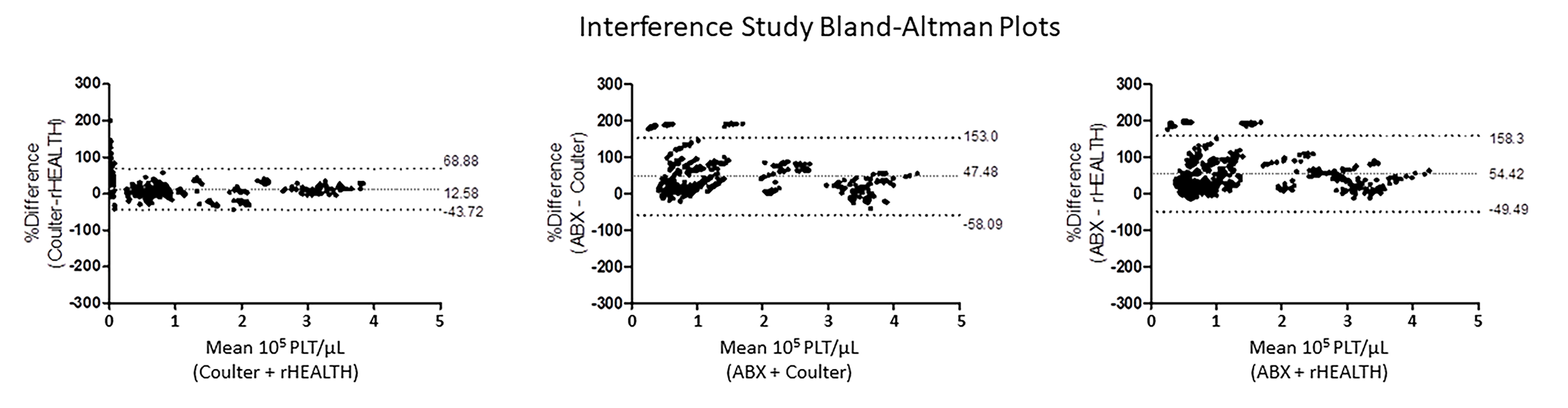

Supplement: S2 Fig — The bias is shown with +/- 1.96 SD bounds. Left-to-right, Coulter ISLH versus rHEALTH, ABX versus Coulter ISLH, and ABX versus rHEALTH. Thrombocytopenia is < 1.5x10^5^ PLT/μL and normal range is 1.5x10^5^ to 4.5x10^5^ PLT/μL. (TIF) [file pone.0256423.s002.TIF]
